# Ethical and Animal Welfare Considerations in Relation to Species Selection for Animal Experimentation

**DOI:** 10.3390/ani4040729

**Published:** 2014-12-03

**Authors:** John Webster

**Affiliations:** Emeritus, University of Bristol, Old Sock Cottage, Mudford Sock, Yeovil, Somerset BA22 8EA, UK; E-Mail: john.webster@bristol.ac.uk

**Keywords:** reduction, replacement, refinement, utilitarianism, autonomy, justice, ethical matrix, moral agents, sentience

## Abstract

**Simple Summary:**

When making a choice of species for animal experimentation we must balance its suitability as a model for human medicine against the potential harms to the animals both from the procedures and the quality of their lifetime experience. The capacity to experience pain may be similar in mammals, birds and fish. The capacity to suffer from fear is governed more by sentience than cognitive ability, so it cannot be assumed that rodents or farm animals suffer less than dogs or primates. I suggest that it is unethical to base the choice of species for animal experimentation simply on the basis that it will cause less distress within society.

**Abstract:**

Ethical principles governing the conduct of experiments with animals are reviewed, especially those relating to the choice of species. Legislation requires that the potential harm to animals arising from any procedure should be assessed in advance and justified in terms of its possible benefit to society. Potential harms may arise both from the procedures and the quality of the animals’ lifetime experience. The conventional approach to species selection is to use animals with the “lowest degree of neurophysiological sensitivity”. However; this concept should be applied with extreme caution in the light of new knowledge. The capacity to experience pain may be similar in mammals, birds and fish. The capacity to suffer from fear is governed more by sentience than cognitive ability, so it cannot be assumed that rodents or farm animals suffer less than dogs or primates. I suggest that it is unethical to base the choice of species for animal experimentation simply on the basis that it will cause less distress within society. A set of responsibilities is outlined for each category of moral agent. These include regulators, operators directly concerned with the conduct of scientific experiments and toxicology trials, veterinarians and animal care staff; and society at large.

## 1. Introduction

*“The great fault of all ethics hitherto has been that they believed themselves to have to deal only with the relations of man to man. In reality, however, the question is what is his attitude to the world and all life that comes within his reach.”* 

—Albert Schweitzer

The ethical and legislative principles by which to justify and define good practice with regard to scientific procedures with animals “calculated to cause pain, distress, suffering or lasting harm” have been established within international treaties such as the Amsterdam Treaty Protocol [1], international legislative provisions including the Council of the European Union Convention ETS123 [2], national legislation such as the UK Animals (Scientific Procedures) Act [3] and the European Directive on the protection of animals used for scientific purposes [4]. They require that the potential harms to animals under experiment should be assessed in advance and justified in terms of their possible benefit to the society of humans or (more rarely) other animals. Of course, the harms and benefits do not accrue to the same species and the species to which the harm is done cannot contribute to the decision-making process. These unfortunate facts create a major ethical dilemma, but not one that comes within the remit of this article.

The principles that govern the need to minimise harm are encapsulated within the classic triad of Russel and Burch [5], namely “reduction, replacement, and refinement”; the three R’s. Briefly stated, reduction means using the smallest possible number of living animals to achieve the desired objective. Replacement refers to the use of non-sentient organisms, or direct studies with humans, as an alternative to the use of protected animals for experiments. Refinement has two applications. It refers to any changes in protocol that can reduce the incidence or severity of distress experienced by living vertebrate animals in consequence of scientific procedures. It also refers to any changes in husbandry that can improve their welfare assessed in terms of their lifetime experience. ASPA (1986) interprets the principles of the three R’s as follows: “*When an experiment has to be performed, the choice of species shall be carefully considered and, where necessary, explained to the authority. In a choice between experiments, those which use the minimum number of animals, involve animals with the lowest degree of neurophysiological sensitivity, cause the least pain, suffering, distress or lasting harm and which are most likely to provide satisfactory results shall be selected”.*

The broad aim of my paper is to contribute to the discussion as to how best we may apply ethical principles to the conduct of scientific procedures with animals. Of course, one fundamental principle of moral philosophy is that some harms are unacceptable in any circumstances. However, this paper starts from the premise that the use of animals for procedures necessary to advance science and protect human health and safety does not fall into this category. My specific aim is to examine the ethical and welfare issues that should govern the choice of species within the orders of sentient animals for scientific procedures that may cause pain, suffering distress or lasting harm. The first step should always be to explore alternatives to the use of sentient animals. However, when there is no realistic alternative we need to ask: “How do the potential benefits accruing from the choice of a particular species rank against any changes, for better or worse, in the harm done to the test animals?” This paper will examine the question within the context of key principles and theories of moral philosophy and good husbandry, namely the educated and compassionate care of animals used in scientific procedures. This paper will explore these questions within a formal ethical matrix that seeks to achieve justice for all creatures involved directly or indirectly in scientific procedures with sentient animals. Many of the views expressed in this paper emerged from productive discussion with my colleagues Peter Bollen, Herwig Grimm and Maggie Jennings during the preparation of an earlier paper on the ethical implications of using the minipig in regulatory toxicology studies [6].

## 2. Ethics

Ethics, synonymous with moral philosophy, is a structured approach to examining and understanding the moral life. Classical, or “top down” ethics asks the question “Which general moral norms for the evaluation and guidance of conduct should we accept and why?” Olsson *et al.* [7] discuss this on the basis of three ethical theories, *Contractarianism, Utilitarianism* and *Animal Rights.* Briefly, contractarianism requires humans, as moral agents, to afford an appropriate degree of protection to animals within our care, the moral patients. The morality of this approach is limited by the fact that it is us, not them who decides how much protection is appropriate; e.g., pets are liable to be given more protection than farm animals. Singer [8] has profoundly explored the application to non-human species of the theory of utilitarianism, namely the practice of beneficence and non-maleficence to achieve the greatest good for the greatest number. The extent to which we may do good or harm will be defined by the capacity of the recipient animal (human or non-human) to experience pleasure or suffering. The third theory, that of Animal Rights [9] is typically applied to define boundaries that should not be crossed, e.g., the conferring to primates of the right not to be used in experimentation. It should be clear by the end of my argument that I do not believe this to be a useful argument, not least because the animals to which we seek to confer rights have not contributed to the discussion.

This “top-down” approach makes for good philosophy but can have difficulty dealing with the complexities and uncertainties of real life. Moreover the ethical arguments that we use to justify our actions are of absolutely no concern to the animals. *It is what we do that counts.* An alternative “bottom-up” approach to the ethical evaluation of real-life situations in which we may do both good and harm is first to identify the specific practical issues, then proceed to a step-by step analysis of the relevant moral issues. Beauchamp and Childress [10] have used this approach to address problems in Biomedical Ethics. It is built upon three pillars of common morality defined as “promoting well-being”, utilitarianism (the greatest good for the greatest number), “autonomy”, respect for the individual (“do as you would be done by”); and “justice” which incorporates principles of equality and fairness. I suggest that it is helpful to think of utilitarianism and autonomy as inputs to moral action and justice as a moral outcome. The notion of justice for individual animals used in scientific procedures is illogical since they can derive no direct benefit to offset the harms done. In this context therefore, the concept of justice places the onus entirely on us, the moral agents to do the best we can for our moral patients. It demands, obviously, that we should seek a fair and humane compromise between the likely benefits to humans of specific procedures and their potential to cause pain, suffering, distress or lasting harm to the test animals.

Our second, maybe less obvious but equally important aim should be to do all we can to ensure that the animals enjoy the best possible quality of life at all times when not directly involved in experiments. This is consistent with the principle of autonomy, which implies much more than the absence of suffering and pain. Rollin [11] states: “Not only will welfare mean control of pain and suffering, it will also entail nurturing and fulfilment of the animals’ natures, which I call telos,” (as did Aristotle) “the unique, evolutionarily determined, genetically encoded, environmentally shaped set of needs and interests which characterize the animal in question—the ‘pigness’ of the pig, the ‘dogness’ of the dog, and so on”. There are those, including philosophers [12] who argue that the concept of telos is not well defined and that it is possible to deal satisfactorily with the issues in response to which the concept was evoked without giving up the idea that welfare is all that matters from a moral point of view in our dealings with animals. Personally, I find telos to be a useful concept. However, I agree with Sandoe that it may be more useful simply to state that our aim should be to provide animals with a physical and social environment as satisfactory as possible in terms of their phenotype and experience [13].

## 3. Moral Agents and Moral Patients: The Ethical Matrix

The balancing of harms to animals against benefits to society is the central question in the analysis of experiments with animals but it is not the only one. The ethical approach must be to seek a fair and just compromise between the reasonable expectations of all concerned parties, whether directly or indirectly involved. Table 1 sets out the principles and identifies the concerned parties in the form of an ethical matrix [14]. The three columns identify the three ethical principles, wellbeing, autonomy and justice. The five concerned groups are:

1. Human society at large: the beneficiaries of new science, pharmaceuticals and other substances tested on animals
2. Regulators: those regulating procedures designed for the advancement of science and statutory testing for product safety.
3. Operators: those licensed to carry out scientific procedures with animals, controllers of pharmaceutical and testing companies, suppliers of test animals.
4. Animal care staff: technicians and veterinarians directly concerned with animal care
5. Experimental and breeding animals

**Table 1:** Application of the ethical matrix to the use of animals in scientific procedures, regulatory toxicology and drug testing.

	Wellbeing	Autonomy	Justice
**Human society at large**	Improved health Product safety	Freedom of choice among available therapies and products	Compassionate and informed recognition of the harms to the test animals
**Regulators of products Regulators of animal experiments**	Responsibility to society (health and safety) Responsibility to animals (minimise harms)	Open-minded approach to new developments (e.g., testing methods)	Respect for animal welfare enshrined in legislation and codes of practice
**Operators(Scientists, pharmaceutical industry, animal breeders)**	Financial reward Informed and sympathetic regulation of procedures	Open-minded approach to new developments (e.g., testing methods).	Compassionate interpretation of legislation.Apply three R’s
**Animal care staff**	Pride and security in work	Control over decisions: e.g., animal husbandry and end-points	Input into animal welfare policy
**Experimental animals**	Minimal harm from procedures Physical and emotional well-being through good husbandry	Environmental enrichment	Just interpretation of harm:benefit equation

The first four groups are all moral agents, the animals are the moral patients. The Ethical Matrix as set out in Table 1 presents a structure for discussion of the ethical issues. The phrases within each of the boxes are simply headlines. It is not possible within the scope of this paper to consider all the responsibilities of the various moral agents. However, I offer a few examples to illustrate how the matrix can be made to work. It is (for example) self-evident that improved scientific knowledge, health and product safety contribute to the well-being of society at large (Group 1) and, of course, to the welfare of other domestic animals. It is generally, though not universally accepted that the properly regulated practice of scientific procedures with animals is essential to this aim (Groups 2 and 3). Equally we recognise that financial success and pride in work are proper elements of wellbeing. This applies both to scientists and staff with day-to-day responsibility for animal care (Groups 3 and 4). However, these “rights” bring responsibilities. The utilitarian principle of respect for animal wellbeing relates, of course, to the principle of minimising harm directly associated with scientific procedures, whether for the advancement of knowledge or for toxicity testing. This applies not only to the physical effects of the procedures themselves but also to any emotional effects of the procedures (including handling and restraint) and other aspects of the animals’ lifetime experience. It therefore requires that proper attention should be given to the physical and emotional welfare of all laboratory animals from their birth to death. Utilitarian principles dictate that the general wellbeing of society depends on scientists who seek new knowledge and regulators who demand product testing and trials with animals to ensure public health and safety. The principle of justice requires that the regulators ensure that proper respect for the test animals is enshrined in legislation and codes of practice, and that licensed experimenters and animal care staff ensure that these principles are implemented in practice.

Two practical expressions of the principle of autonomy, as it applies to society at large, are competition and freedom of choice, both of which are encouraged through the development of new, desirable drugs and chemicals. The principle of autonomy, as applied to regulators and operators of animal experiments implies that both parties should be open-minded to new ideas. This is particularly important in the case of toxicology testing where it can be too easy to stick to long-standing methods and choice of species, because “we have always done it this way”.

Any discussion of the ethics of animal experimentation must include the application of the three principles to the animal care staff. They have the right to enjoy pride and security in their work. To this end they should be given every encouragement to ensure the best possible welfare for the animals in their care, not only on a day-to-day basis but also through opportunities to contribute to strategic decisions, e.g., in regard to housing and environmental enrichment and setting end-points for procedures calculated to cause chronic suffering or lasting harm.

Application of the utilitarian principle to the experimental animals requires the need to minimise harm from the procedures themselves and to promote physical and emotional well-being through good husbandry on a lifetime basis. Autonomy can be encouraged through the design and provision of enriched environments that provide freedom of choice for individuals without compromising the welfare of others. The outcome, justice for the animals, requires that all who work with experimental animals, who commission work with experimental animals, or who benefit from the outcome of such work, should promote their welfare and minimise harms through policies based upon the principle of respect for all life.

## 4. Species Selection: Ethical Issues and Practical Questions

The ethical matrix provides a framework upon which to identify and explore issues raised by the moral imperative to seek a fair compromise between the differing needs of the different interest groups (Table 1). We should seek the most humane solution to the harm/benefit assessment for every class of experimental animal and every procedure. In the specific context of species selection we are faced with four key questions.

1. How do species compare as models for the physiological, psychological or medical function in humans that the experimental procedure seeks to reproduce?
2. To what extent may different classes of sentient animal (e.g., fishes, birds and mammals) or different species within the class *mammalia* (e.g., rodents, farm animals, dogs and primates) differ (or not) in their capacity for pain and suffering?
3. To what extent may the choice of species reduce the harm associated directly or indirectly with a specific scientific or testing procedure?
4. To what extent is, or should the choice of species be encouraged or constrained by human values that are unsupported by scientific evidence?

Questions 1 and 2 are matters of science and practical expediency. This implies that the answers are not fixed and should always be subject to further questioning and revised in the light of new understanding. Question 3 arises from the moral need to minimise harms and involves both science and ethics. Question 4 is entirely a matter of ethics.

## 5. Species Suitability as Models for Human Physiology and Medicine

Considered simply in terms of human self-interest, the suitability of a species for procedures regulated by the EU Directive 2010/63 [4] is likely to be determined by its assumed similarity to human physiology, pathophysiology, psychology or behaviour. Other important considerations include convenience and cost, and the predictability and consistency of the outcome measures of the trial, based on past experience and the genetic uniformity of the test animals. Genetically similar or cloned rats and mice have obvious advantages in all these respects. It is not possible to come to any general conclusions regarding the relative suitability of different non-rodent species as models for humans. Webster *et al.* [6] have reviewed factors affecting choice between minipigs, dogs and primates. For example, in some toxicological or immunological studies, a valid case can be made for the use of the minipig because its skin is similar to humans. The dog has been chosen as the preferred species in the development of anti-ulcer drugs, since dogs have a similar gastric mucosal membrane to that of humans. A consensus is emerging within the scientific community that the use of primates should be restricted to those experiments for which there is, at this time, no known alternative [15,16]. This could restrict the choice of primates to certain studies in neuroscience and brain function and communicable diseases common to man and other primates (e.g., HIV/AIDS and tuberculosis). Some important questions, e.g., many studies of the immune system, are so species-specific that no non-human species can serve as an ideal predictive model. However, the problem is being addressed by new science. For example, Geertje *et al.* [17] have demonstrated that minipigs are no less effective than primates as a model for human immunogenicity testing.

All the arguments outlined above can and should be incorporated into the decision as to the selection of the most suitable species for a specific procedure. However, choice of species should never be made on the basis of conservatism. The continued use of dogs and primate species as non-rodent subjects in regulatory toxicology is often justified by the preamble “the species has been used in the past, there is a substantial library of knowledge and it is acceptable to the regulators”. Obviously the decision as to whether or not use dogs or primates for scientific and regulatory procedures will involve factors other than their suitability as models for human function. These other factors are discussed later. At this stage it is sufficient to say that the continued use of these two species must be justified by state-of-the-art science, not by tradition.

## 6. Sentience and “Neurophysiological Sensitivity”

ASPA defines the rules of engagement for the protection of “any living vertebrate other than man” when used in procedures likely to cause **“**pain, suffering, distress or lasting harm**”**. EU Directive 2010/63/EU states “in addition to vertebrates, including cyclostomes, cephalopods should also be included in the scope of this Directive, as there is scientific evidence of their ability to experience pain, suffering, distress and lasting harm”. However, ASPA, while affording protection to all these animals, then proceeds to state that the aim should be to “involve animals with the lowest degree of neurophysiological sensitivity”. This begs the question ‘Can we be sure of our assumptions as to differences in neurophysiological sensitivity between classes of vertebrates (e.g., mammals, birds and fish), still less between species of mammals (e.g., mice, rats, pigs, dogs and primates)?”.

Let us consider first the matter of pain. It is now beyond cavil that, for all mammals, pain is a physical and emotional experience. It is much more than just an unpleasant sensation; it also induces changes in behaviour and mood broadly similar to those seen in humans [18]. Evidence is accumulating to indicate that similar responses to pain are seen in birds [19] and fish [20]. In respect to procedures calculated to cause pain it would appear to be unjust to distinguish between these three classes of sentient animal in terms of neurophysiological sensitivity.

It is valid, however, to ask whether different classes of sentient animal, or different species with the class *mammalia* differ in their response to scientific and husbandry procedures in elements of suffering other than pain, especially those elements with the potential to cause long-term psychological distress (e.g., anxiety). I have previously defined a sentient animal as one that has **“**feelings that matter” [13]. To expand this definition: sentient animals interpret sensations and experiences primarily in emotional terms and are motivated to behaviour designed to make them feel good and avoid feeling bad. This emotional basis to motivation may, or may not, be modified by cognition (or reason). Having behaved in a way designed to achieve a favourable physical and emotional state, the animal reviews the consequences of its actions. If these are successful, it will achieve a sense of well-being because it has learned to cope. If it fails to cope it is liable to suffer distress. It follows from this that all sentient mammals can suffer distress not only from consequences of scientific procedures, such as pain, fear and malaise, but also from the emotional consequences of failure to cope; *i.e.*, failure to achieve their physiological and behavioural needs within the constraints of their environment. Such long-term consequences cover a spectrum of distress that ranges from chronic anxiety to learned helplessness. Thus means that the capacity of a sentient animal to suffer is defined by its emotional potential, and not necessarily by its cognitive ability. Cognitive understanding of causes and consequences can make things either better or worse. Consider, for example, the effect of human consciousness on the interpretation of pain from dental surgery or stomach cancer. Webster *et al.* [6] have argued on this basis that it cannot be assumed that a rat or pig is less (or more) capable of suffering in any form than a primate simply on the basis that it appears to be “less like us” in terms of neurophysiological sensitivity.

## 7. Minimising Harms

The responsibility of those who work with animals used for scientific purposes, toxicology and drug testing is directed, in the first instance, to minimising harms associated with all scientific procedures calculated to cause pain, suffering, distress or lasting harm, e.g., infections, induced fractures, genetic modifications known to be associated with significant abnormalities. However, our responsibility should also embrace a proper concern for the lifetime welfare of the animals based on a professional understanding of the special physiological, behavioural and emotional needs of the species concerned. These include:

- Comparison of harms, both physical and emotional, associated with direct interference with the animals in the course of scientific procedures: e.g., blood sampling, gavage *etc.* and the restraint involved with such procedures.
- Adequacy of knowledge and procedures for assessment of pain and distress and identification of humane end points.
- Quality of housing and husbandry for test, stock and breeding animals based, wherever possible on the principle of environmental enrichment.
- Quality of animal care based on a competent and compassionate understanding of the human/animal bond as it applies to the different species.

In all these cases, the effectiveness of practical steps to reduce harm and to enrich lifetime experience, will depend on the professional competence and compassionate understanding of those responsible for day-to-day care of the animals *and* those responsible for the design and strategic management of the laboratory. Moreover, much of this will need to be species-specific. For example, it may be less stressful to handle a dog than a pig during experimental procedures, but a dog may well display greater anxiety either in anticipation of future procedures or during separation from the animal care staff. The behavioural indices of pain are also very species-specific. Recognition of pain in sheep, a stoic, stone-faced species, requires knowledge of some very subtle signals [21].

## 8. Application of the Three R’s

**Refinement**: Substituting a “lower” species (phylogenetic reduction), is often considered to be refinement. Indeed it is encapsulated in the guidance to ASPA (1986) namely to choose animals with the “*lowest degree of neurophysiological sensitivity”.* However, it should be clear by now that I believe this cannot be assumed simply on phylogenetic grounds. Such a judgement can only be made if assessment of the available scientific evidence suggests that the lower species is less sentient and therefore less likely to suffer. In most cases the scientific evidence does not exist, but where it does, e.g., pain in fish [20] it indicates that we should not act on such assumptions in the absence of definitive proof. Indeed the more evidence we accumulate, the more we should be aware of the limits of our understanding and the need to give the “lower” species the benefit of the doubt. Judgements about whether it is more humane to use one species over another are particularly difficult where the species are closely related phylogenetically (e.g., species within the class *Mammalia* or order Primates). I contend that it is not possible to distinguish *a priori* between sentient mammalian species in terms of their capacity to suffer physical or emotional harm. It is however, possible to make inferences about the likely relative impact of different procedures and husbandry practices on the welfare of different species from what is known from published evidence and practical experience about their natural history, behaviour and welfare needs (e.g., importance of companionship, response to laboratory housing, habituation to humans) and the potential stressors involved with their use (e.g., capture from the wild, long and multi-staged transport, degree of restraint required). Decisions as to species selection should be based on an assessment of the sum total of harms involved after all the relevant information has been taken into account.

## 9. Reduction

Death is, by any definition, a lasting harm. It is thus morally right to seek to reduce the number of animals bred, and subsequently killed, in laboratories irrespective of whether or not they have suffered during life. The use of inbred, specific pathogen free (SPF), or genetically modified (GM) animals can, in some circumstances, lead to the need for smaller sample sizes in scientific procedures [22]. Species selection can impact on reduction in various ways. For example, choice of the most appropriate species in terms of applicability to human physiology and medicine might help to avoid the need to repeat studies in the future and, in so doing, avoid use of additional animals. Use of a well-characterised species instead of a less familiar one may also permit reduction of animal numbers. Use of a species with large litters (e.g., rodents, minipigs) may permit reduction of the total number of animals required for a given study, e.g., reduction of the numbers of animal mothers if each infant in the litter (rather than each litter) is considered to be an ‘experimental unit’. In the matter of toxicology, this applies particularly to “Reprotox” tests; studies of the impact of test substances on the offspring of the challenged animal.

## 10. Traditions and Other Values Unsupported by Scientific Evidence

It may be argued that, according to common morality, it is more acceptable to use rodents or farm animals such as the pig in preference to dogs or primates for scientific procedures or toxicity testing on the basis that this will cause less distress within society. The pros and cons of this argument have been reviewed by Hobson-West [23]. Some countries afford special protection to certain higher mammals in their legislation, reflecting increased public concern for these species. For example, ASPA states that: “*The Secretary of State shall not grant a project licence authorising the use of cats, dogs, primates or equidæ unless he is satisfied that animals of no other species are suitable for the purposes of the programme to be specified in the licence or that it is not practicable to obtain animals of any other species that are suitable for those purposes.”* Just application of the Ethical Matrix (Table 1) requires us to show proper concern for all concerned parties, which include the animal care staff who might find it less distressing to carry out studies with rodents or pigs than with dogs or primates. If this relative lack of concern were based on an impression that species that we do not class as companion animals are somehow less sentient, and thus less likely to suffer than those we have chosen to love, it would be invalid. Thus the animal care staff needs to be helped to understand that the capacity of a species to experience suffering and pleasure is determined entirely by its own sentience and not by their status in human society.

It is important to remember, however, that species-specific sentience does incorporate the nature of the human/animal bond. Webster *et al.* [6] suggested that animals such as minipigs, maintained to the highest standards of care in research laboratories, might be less likely to develop close personal bonds with individual members of the animal care staff than selected breeds of dog (e.g., Beagles) or species of monkey. If this were shown to be so, one could make a valid argument for choosing a species such as the pig or sheep in preference to dogs or primates on the basis that this might reduce anxiety and stress both for experimental animals and animal care staff. Nevertheless, I repeat my fundamental assertion that substitution of one mammalian species for another does not constitute replacement within the context of the three R’s. Therefore I cannot accept the premise that species selection for scientific procedures with animals can be made simply on the grounds that it may prove less offensive to some groups within society at large.

## 11. Responsibilities and Regulation

The ethical concerns outlined above confer significant responsibilities on the many concerned parties: the four categories of moral agent, as outlined and structured by the Ethical Matrix (Table 1), namely Regulators, Operators, Animal Care Staff and Society at large. A full list of these responsibilities is beyond the scope of this paper. In the specific context of species selection, the **regulators** (e.g., legislators, regulatory authorities) and senior operators (e.g., directors of pharmaceutical companies) should ensure the following:

- Regular review of procedures in the context of the harm/benefit equation so as to inform future decisions in relation to choice of species for specific procedures and husbandry appropriate to that species.
- That the operators involved in breeding animals and/or carrying out procedures have the facilities, sufficient resources and staff with training specific to the species, to carry out their work with efficiency and humanity.
- That the suitability of the species for each procedure (by ranking benefits and harms) is justified by state-of-the–art knowledge rather than habits engrained by custom and tradition.

The **operators**, licensed scientists, veterinarians and animal-care staff have the responsibility to develop a compassionate and professionally competent approach to the welfare of animals under their care based on a profound understanding of the physiological, behavioural and emotional needs of the species involved. The animal care staff must have the ability to recognise early signs of physical or mental suffering, know what to do about this and/or who else to contact to communicate any animal welfare concerns to those whose prime responsibility is the successful outcome of the trials, to ensure that the welfare of the animals is not unduly compromised. The scientists have the responsibility to respect this advice and act accordingly.

**Society at large**, the patients and consumers who benefit from scientific and regulatory procedures with animals have the right to expect that medicines and other products have been subjected to satisfactory test procedures. However, this carries responsibilities: to seek information about the way drugs are developed and tested, to accept that absolute safety is not possible and so not exacerbate the demand for excessive testing and especially, in the context of this paper, to avoid assumptions about the acceptability of animal use based on the appeal of the species to them, rather than its own sentience.

## References

[1] (1997). Animal Welfare and the Treaty of Amsterdam. http://www.eurocbc.org/page673.html.

[2] Council of the European Union (1986). European Convention for the Protection of Vertebrate Animals Used for Experimental and Other Scientific Purposes. ETS 123. http://conventions.coe.int/treaty/en/treaties/html/123.htm.

[3] (1986). Animals (Scientific Procedures) Act. http://www.legislation.gov.uk/ukpga/1986/14/contents.

[4] (2010). Directive of the European Parliament and of the Council of 20 September 2010 on the Protection of Animals Used for Scientific Purposes.

[5] Russel W.M., Burch R.L. (1959). The Principles of Humane Experimental Technique.

[6] Webster J., Bollen P., Grimm H., Jennings M. (2010). Ethical implications of using the minipig in regulatory toxicology studies. J. Pharmacol. Toxicol. Methods.

[7] Olsson I.A.S., Robinson P., Sandøe P. (2010). Handbook of Laboratory Animal Science.

[8] Singer P. (1977). Animal Liberation. Towards an End to Man’s Inhumanity to Animals.

[9] Regan T., Regan T., Singer P. (1989). The Case for Animal Rights. Animal Rights and Human Obligations.

[10] Beauchamp T.L., Childress J.F. (2001). Principles of Biomedical Ethics.

[11] Rollin B.E. (1993). Animal Welfare: Science and Value. J. Agric. Environ. Eth..

[12] Sandøe P., Hocking P.M., Forkman N., Haldane K., Kristensen H.H., Palmer C. (2013). The blind hens’ challenge: Does it undermine the view that only welfare matters in our dealings with animals?. Environ. Values.

[13] Webster J. (2005). Animal Welfare: Limping towards Eden.

[14] Mepham B. (2000). A Framework for the Ethical Analysis of Novel Foods: The Ethical Matrix. J. Agric. Environ. Eth..

[15] Boyd Group (2002). The Use of Non-Human Primates in Research and Testing.

[16] (2006). The Weatherall Report on the Use of Non-Human Primates in Research.

[17] van Mierlo G.J., Cnubben N.H., Kuper C.F., Wolthoorn J., van Meeteren-Kreikamp A.P., Nagtegaal M.M., Doornbos R., Ganderup N.C., Penninks A.H. (2013). The Göttingen minipig^®^ as an alternative non-rodent species for immunogenicity testing: A demonstrator study using the IL-1 receptor antagonist anakinra. J. Immunotoxicol..

[18] Morton D.B., Griffiths P.H. (1985). Guidelines on the recognition of pain, distress and discomfort in experimental animals and an hypothesis for assessment. Vet. Record.

[19] Danbury T.C., Weeks C.A., Chambers J.P., Waterman-Pearson A.E., Kestin S.C. (1999). Self-selection of the analgesic drug carprofen by lame broiler chickens. Vet. Record.

[20] Ashley P.J., Sneddon L.U., Branson E.J. (2008). Pain and Fear in Fish. Fish Welfare.

[21] Anil S.S., Anil L., Deen J. (2002). Challenges of pain assessment in domestic animals. J. Am. Vet. Med. Assoc..

[22] Festing M. (2004). The choice of animal model and reduction. Altern. Lab. Anim..

[23] Hobson-West P. (2010). The role of ‘public opinion’ in the UK animal research debate. J. Med. Eth..

